# Characteristic Changes of the Stance-Phase Plantar Pressure Curve When Walking Uphill and Downhill: Cross-Sectional Study

**DOI:** 10.2196/44948

**Published:** 2024-05-08

**Authors:** Christian Wolff, Patrick Steinheimer, Elke Warmerdam, Tim Dahmen, Philipp Slusallek, Christian Schlinkmann, Fei Chen, Marcel Orth, Tim Pohlemann, Bergita Ganse

**Affiliations:** 1 German Research Center for Artificial Intelligence (DFKI) Saarbrücken Germany; 2 Department of Trauma, Hand and Reconstructive Surgery Departments and Institutes of Surgery Saarland University Homburg/Saar Germany; 3 Innovative Implant Development (Fracture Healing) Departments and Institutes of Surgery Saarland University Homburg/Saar Germany

**Keywords:** podiatry, podiatric medicine, movement analysis, ground reaction forces, wearables, slope, gait analysis, monitoring, gait, rehabilitation, treatment, sensor, injury, postoperative treatment, sensors, personalized medicine, movement, digital health, pedography, baropedography

## Abstract

**Background:**

Monitoring of gait patterns by insoles is popular to study behavior and activity in the daily life of people and throughout the rehabilitation process of patients. Live data analyses may improve personalized prevention and treatment regimens, as well as rehabilitation. The M-shaped plantar pressure curve during the stance phase is mainly defined by the loading and unloading slope, 2 maxima, 1 minimum, as well as the force during defined periods. When monitoring gait continuously, walking uphill or downhill could affect this curve in characteristic ways.

**Objective:**

For walking on a slope, typical changes in the stance phase curve measured by insoles were hypothesized.

**Methods:**

In total, 40 healthy participants of both sexes were fitted with individually calibrated insoles with 16 pressure sensors each and a recording frequency of 100 Hz. Participants walked on a treadmill at 4 km/h for 1 minute in each of the following slopes: −20%, −15%, −10%, −5%, 0%, 5%, 10%, 15%, and 20%. Raw data were exported for analyses. A custom-developed data platform was used for data processing and parameter calculation, including step detection, data transformation, and normalization for time by natural cubic spline interpolation and force (proportion of body weight). To identify the time-axis positions of the desired maxima and minimum among the available extremum candidates in each step, a Gaussian filter was applied (σ=3, kernel size 7). Inconclusive extremum candidates were further processed by screening for time plausibility, maximum or minimum pool filtering, and monotony. Several parameters that describe the curve trajectory were computed for each step. The normal distribution of data was tested by the Kolmogorov-Smirnov and Shapiro-Wilk tests.

**Results:**

Data were normally distributed. An analysis of variance with the gait parameters as dependent and slope as independent variables revealed significant changes related to the slope for the following parameters of the stance phase curve: the mean force during loading and unloading, the 2 maxima and the minimum, as well as the loading and unloading slope (all *P*<.001). A simultaneous increase in the loading slope, the first maximum and the mean loading force combined with a decrease in the mean unloading force, the second maximum, and the unloading slope is characteristic for downhill walking. The opposite represents uphill walking. The minimum had its peak at horizontal walking and values dropped when walking uphill and downhill alike. It is therefore not a suitable parameter to distinguish between uphill and downhill walking.

**Conclusions:**

While patient-related factors, such as anthropometrics, injury, or disease shape the stance phase curve on a longer-term scale, walking on slopes leads to temporary and characteristic short-term changes in the curve trajectory.

## Introduction

Long-term monitoring of gait patterns and plantar-pressure distributions via insoles are increasingly popular ways to study behavior and activity in the field and in the everyday lives of people and patients, including healing, personalized prevention, and treatment or disease progression [1-3]. In recent years, the usability of instrumented insoles for gait analyses has increased. Several technical issues could be resolved, including calibration, hysteresis and drift, durability, usability, limited energy supply and battery life, data storage capacity, and the restriction to low sample frequencies associated with higher error rates, that is, when force peaks are missed [3-5]. The usability of instrumented insoles is currently still limited by difficulties in data analysis. Advanced algorithms and tools are needed and currently developed to be able to draw meaningful conclusions from such insole gait data [6,7]. When analyzing long-term field data and developing smart health care innovations, automated data annotation is desirable to determine and quantify the activities a person has conducted. Ideally, the activity type can be determined algorithmically from the plantar pressure data alone.

Characteristic gait changes have been reported for walking on slopes, such as changes in the contribution of the ankle joint to leg work [8]. In addition, uphill walking on a treadmill increases hip and knee flexion angles during the stance phase, as well as the forward tilt of the thorax [9]. Furthermore, a decrease in dorsiflexion was observed during downhill walking at initial contact, in midstance, and during the second half of the swing phase [9]. During uphill walking with increasing inclination, more positive joint work was identified for the ankle and hip joint, while negative joint work increased during downhill walking [10]. Older individuals were shown to have a disproportionate recruitment of hip muscles and smaller increases in activity of the medial gastrocnemius muscle with steeper uphill slopes than younger adults, resulting in difficulty walking on steep slopes [11].

The M-shaped curve of ground reaction forces or plantar pressure during the stance phase is mainly defined by the loading and unloading slope, 2 maxima, 1 minimum, as well as the force during defined periods [12]. When monitoring gait continuously via insoles, walking uphill or downhill on a slope could affect the gait cycle curve in characteristic ways. If these typical changes were known, one could correct for such confounders when analyzing insole data. We hypothesized that walking on a slope generates typical changes in the plantar pressure stance phase curve that vary between uphill and downhill walking.

## Methods

### Study Design

This study is part of the project Smart Implants 2.0—Weight-bearing and Gait Observation for Early Monitoring of Fracture Healing and Individualized Therapy after Trauma, funded by the Werner Siemens Foundation. It was registered in the German Clinical Trials Register (DRKS00025108).

### Ethical Considerations

Ethical approval was obtained from the Institutional Review Board of Saarland Medical Board (Ärztekammer des Saarlandes, 30/21).

### Data Collection

Inclusion criteria were the ability to walk on a treadmill, and aged 18 years and older. Exclusion criteria were aged under 18 years, use of walking aids, inability to give consent, pregnancy, immobility, and previous injury of the lower legs or pelvis. The aim was to collect data from healthy volunteers.

The healthy participants of both sexes (none of them identified as diverse) were fitted with individually calibrated OpenGO insoles (Moticon GmbH) with 16 pressure sensors in each insole to be used in regular running shoes. Calibration to the individual body weight was conducted using the Moticon OpenGO app by letting the participants walk and shift their body weight in a standardized way. The insole size was selected to fit the individual participant’s shoe size. Measurements were conducted with a recording frequency of 100 Hz in the record mode of the device. Raw data were exported for analyses. The participants walked on a treadmill at 4 km/h (Mercury, HP Cosmos) for 1 minute while insole data were collected with 3-minute breaks for recovery. Recordings were obtained for slopes of −20%, −15%, −10%, −5%, 0%, 5%, 10%, 15%, and 20%. The participants were asked to walk for 1 minute straight, and recording was only commenced when the walking was already in progress to avoid bias by including altered steps upon gait initiation.

### Data Processing

The pressure readings of the force sensors in the insole device yield a weighted sum as a total vertical ground reaction force reading. To compute the force, every summand is weighted by its sensor area and a respective scaling factor accounting for the sensor’s surrounding area, as well as gaps between sensors that depend on the insole size. This process is conducted by the Moticon software as an automated processing step before file export. Insole data were exported as described previously [13,14]. A custom-developed data platform was then used for further processing and parameter calculation, in which step detection was conducted as follows. The stance phases were identified and extracted from the time series data by considering any activity with consecutive force readings above 30 N. A tolerance of up to 3 missing values was implemented to account for possible recording issues. Any activity with a duration of less than 300 milliseconds or more than 2000 milliseconds was discarded. Both the force and time axes were normalized. Force readings were transformed from Newton to a proportion of the body weight of the respective participant. Of note, as plantar pressure was measured instead of weight, due to acceleration, values regularly exceeded the body weight for peak load-bearing instances. Normalizing the time axis was more complex, as the lack of a fixed cadence resulted in varying step lengths and thus differing numbers of true measurement points for each step. Therefore, a natural cubic spline interpolation was conducted on the original raw data. Based on the resulting curve for each stance phase, 100 equidistant samples were taken, resulting in an interpolated force measurement point for every 1% of the overall stance phase length. This approach accounted for the lower recording frequency and higher sensor noise inherent to the insoles when compared with other gait measuring devices, such as sensor-equipped treadmills or force plates. Parameters that describe the trajectory of the stance phase curve are usually based on or derived from the characteristic local extrema, that is, the first and second force peak and the local minimum in-between force peaks. These maxima and the minimum are used as parameters themselves to describe the curve trajectory [13]. Sensor jitter may lead to the existence of multiple ambiguous candidates for the named extrema. As a solution to this, a Gaussian filter was applied to the original raw data in a repetition of the normalization process. The applied filtering strategy (σ=3, kernel size 7) was chosen to prioritize the elimination of extrema ambiguity at the expense of signal precision. This can result in overcorrection in areas with higher signal volatility, mostly at the start and end of the stance phase. Hence, to avoid loss of high-frequency detail, the filtered and normalized curve was not used for parameter analysis, but only to determine unambiguous time-axis positions (indices) for the extremum candidates. These indices were then reapplied to the nonfiltered, normalized data to identify the corresponding plantar pressure measurement closest to the original raw data. In case the use of the filtered data still led to inconclusive extremum candidates, the following additional detection strategies were applied in the named order: (1) time plausibility: extremum candidates occurring within the first or last 10 indices (first/last 10% of overall time span) were eliminated; (2) maximum or minimum-pool filtering: should multiple extremum candidates occur within a pool size of 5 indices (equals to 5% of overall time span), the candidate with the highest or lowest force value was chosen; (3) monotony-check: in case of multiple remaining extremum candidates, candidates where the curve did not display a strict monotonous decrease or increase in both directions within 5 indices each were eliminated; and (4) monotony grace: in case the monotony check had eliminated too many candidates (less than 2 maximum candidates or less than 1 minimum candidate remaining), the eliminated candidates were reinstated in descending order of their highest achieved monotony distance until the target number of candidates was reached.

After applying these strategies, every stance activity that remained with an irregular amount of unambiguous extremum candidates was removed from the data set. In total, 585 load-bearing events were excluded as not fitting the strict parameter definitions.

### Parameters

**Figure 1:**
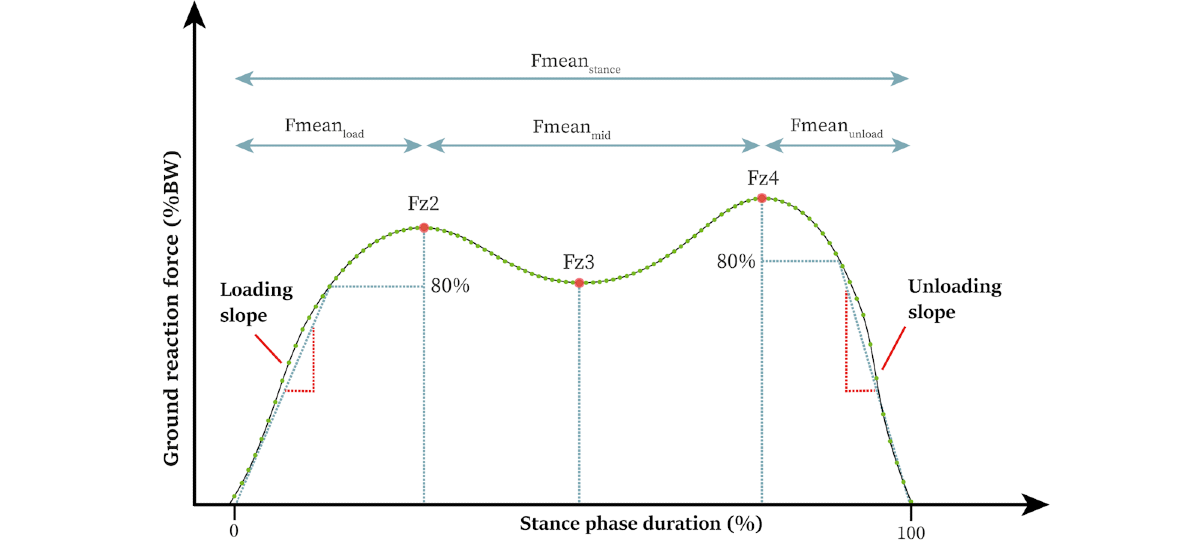
Depiction of the analyzed parameters of the stance phase. %BW: percent body weight; Fmean_load_: the mean force between the start of loading-phase and Fz2; Fmean_mid_: the mean force between Fz2 and Fz4; Fmean_stance_: the mean force over the entire stance phase; Fmean_unload_: the mean force between Fz4 and the end of the unloading-phase; Fz2: the first maximum; Fz3: the minimum; Fz4: the second maximum.

For each participant, across the minute of walking all stance phase curves were extracted. The parameters illustrated in Figure 1 were calculated for each stance phase and used to analyze changes in the trajectory of the stance phase curve. To do so, data from both feet were pooled. The curve is mainly described by 2 maxima and a minimum in between the maxima, Fz2 (the first maximum), Fz3 (the minimum), and Fz4 (the second maximum). The mean force over the entire stance phase is referred to as Fmean_stance_. The mean force between the start of the loading phase and Fz2 is Fmean_load_. The mean force between Fz2 and Fz4 is Fmean_mid_. The mean force between Fz4 and the end of the unloading phase is Fmean_unload_. All these parameters have the unit percent body weight. In addition, the loading and unloading slope have the units percent body weight or percent stance phase duration. The loading slope was computed as the slope of the line defined by the start of the loading phase and the first force reading equal to or higher than 80% of Fz2. The unloading slope was calculated as the slope of the line defined by the first force reading in the unloading phase below 80% of Fz4 and the end of the stance phase event.

### Statistical Analyses

Statistical tests were executed with SPSS Statistics (version 29; IBM Corp). Significance was defined as *P*<.05. The normal distribution of data was tested by the Kolmogorov-Smirnov and Shapiro-Wilk tests. A linear regression analysis of variance was conducted for each of the gait parameters as the dependent variable, with the slope (−20% to 20%) as the independent variable. Mean values and SD are reported. Linear regression slopes are reported for comparability and to allow for correction, even though for some of the parameters other but differing regression types yielded higher *R*^2^ values. The sample size of 40 was an estimate based on what is common in the field, and taking into account the aim to measure a very diverse group of volunteers. An a priori sample size calculation was not conducted due to a lack of comparable data.

## Results

Measurements were taken from 40 healthy participants (19 women and 21 men) with an average age of 43.90 (SD 17.30, range 18-87) years. Participant characteristics are summarized in Table 1. Data were successfully recorded for all of the participants and slope levels, resulting in a complete data set (Multimedia Appendix 1).

**Table 1:** Participant characteristics.

	Total (N=40)	Women (n=19)	Men (n=21)
Age (years), mean (SD)	43.90 (17.30)	39.05 (14.65)	48.29 (18.64)
Height (cm), mean (SD)	174.43 (11.24)	165.79 (6.05)	182.24 (8.85)
Weight (kg), mean (SD)	80.40 (26.85)	66.22 (16.15)	93.24 (28.40)
BMI (kg/m^2^), mean (SD)	23.04 (6.83)	20.15 (5.06)	25.65 (7.28)

**Figure 2:**
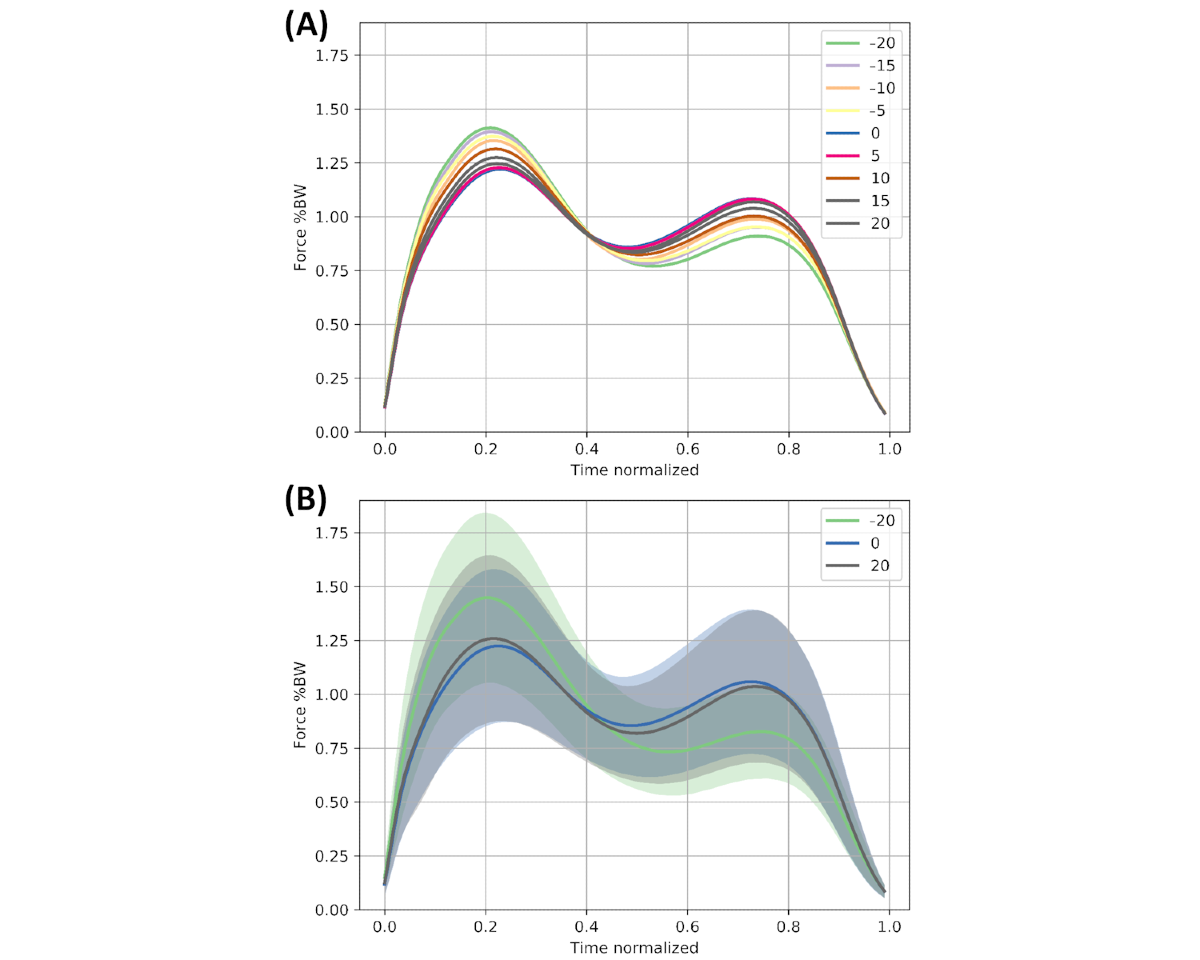
(A) The mean trajectories of the stance phase curve for each of the analyzed slope levels. (B) The mean trajectories and the 95% CI for −20%, 0%, and 20%. %BW: percent body weight.

**Figure 3:**
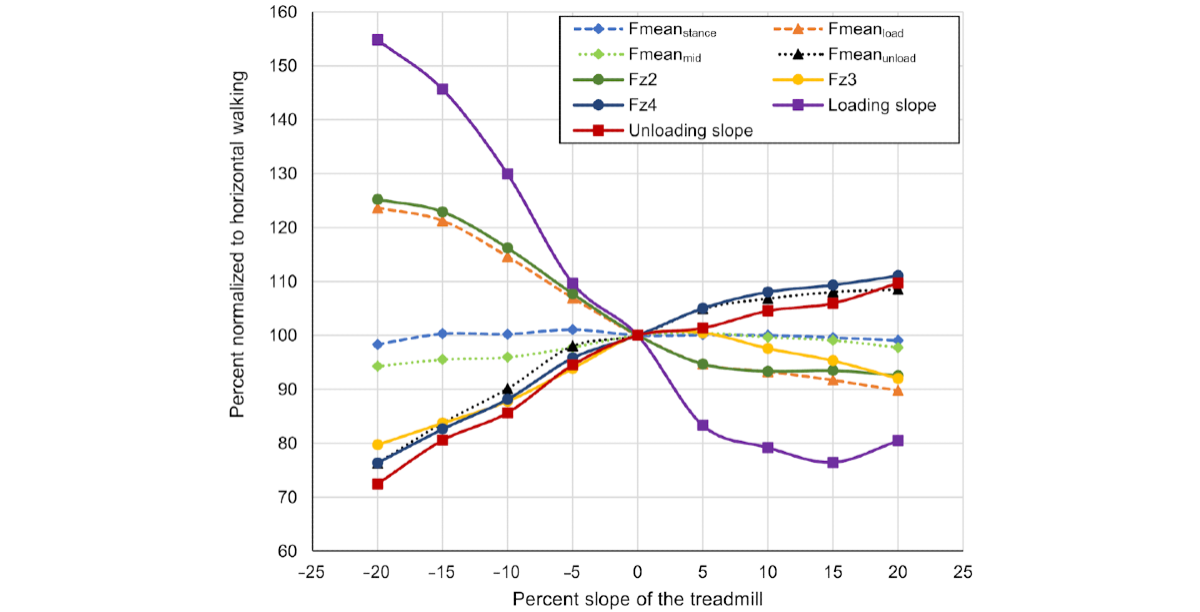
For each slope level, the mean value of each parameter is shown in the percent of horizontal walking (all values averaged over all participants). Fmean_load_: the mean force between the start of loading-phase and Fz2; Fmean_mid_: the mean force between Fz2 and Fz4; Fmean_stance_: the mean force over the entire stance phase; Fmean_unload_: the mean force between Fz4 and the end of the unloading-phase; Fz2: the first maximum; Fz3: the minimum; Fz4: the second maximum.

Data were normally distributed. Figure 2 visualizes the differences between the analyzed slope values on the stance phase curve. Figure 3 shows the normalized changes in the analyzed parameters with the slope of the treadmill. The analysis of variance revealed significant changes with the slope for Fmean_load_, Fmean_unload_, Fz2, Fz3, Fz4, loading and unloading slope (all *P*<.001). There was no significant correlation of the slope with Fmean_stance_ (*P*=.98) and Fmean_mid_ (*P*=.13). Other than the other parameters with significant changes related to slope, Fz3 had its peak at horizontal walking and values dropped when walking uphill and downhill alike. Thus, a simultaneous and short-term increase in loading slope and Fmean_load_ combined with a decrease in Fmean_unload_, Fz2, Fz4, and the unloading slope indicates downhill walking, while the opposite indicates uphill walking. Fz3 is not a suitable parameter to distinguish between uphill and downhill walking, as its value decreases both when walking uphill as well as downhill. Mean values and the SD of the analyzed parameters for each treadmill slope level in absolute values are displayed in Table 2. Table 3 indicates the linear regression slopes and *R*^2^-values for each of the curves shown in Figure 3.

**Table 2:** Mean values and SD of the analyzed parameters for each slope level (absolute values).

	−20%	−15%	−10%	−5%	0%	5%	10%	15%	20%
Fmean_stance_^a^ (% body weight)	0.88 (0.21)	0.90 (0.21)	0.90 (0.21)	0.91 (0.21)	0.90 (0.21)	0.90 (0.21)	0.90 (0.21)	0.89 (0.20)	0.89 (0.20)
Fmean_load_^b^ (% body weight)	1.06 (0.30)	1.04 (0.28)	0.98 (0.26)	0.91 (0.23)	0.86 (0.21)	0.81 (0.20)	0.80 (0.20)	0.78 (0.19)	0.77 (0.18)
Fmean_mid_^c^ (% body weight)	0.97 (0.24)	0.99 (0.23)	0.99 (0.23)	1.01 (0.23)	1.03 (0.25)	1.04 (0.25)	1.03 (0.25)	1.02 (0.25)	1.01 (0.24)
Fmean_unload_^d^ (% body weight)	0.55 (0.14)	0.61 (0.14)	0.65 (0.16)	0.71 (0.16)	0.72 (0.17)	0.76 (0.18)	0.77 (0.18)	0.78 (0.18)	0.79 (0.18)
Fz2^e^ (% body weight)	1.50 (0.39)	1.48 (0.38)	1.39 (0.36)	1.29 (0.33)	1.20 (0.30)	1.14 (0.29)	1.12 (0.29)	1.12 (0.29)	1.11 (0.29)
Fz3^f^ (% body weight)	0.70 (0.20)	0.74 (0.19)	0.77 (0.18)	0.83 (0.19)	0.88 (0.22)	0.88 (0.21)	0.86 (0.21)	0.84 (0.22)	0.81 (0.19)
Fz4^g^ (% body weight)	0.88 (0.24)	0.96 (0.24)	1.02 (0.26)	1.11 (0.28)	1.16 (0.32)	1.22 (0.33)	1.25 (0.33)	1.26 (0.34)	1.29 (0.35)
Loading slope (% body weight/% stance phase duration)	11.00 (4.58)	10.35 (4.01)	9.23 (3.58)	7.79 (2.99)	7.10 (2.91)	5.92 (2.11)	5.63 (2.07)	5.43 (1.85)	5.71 (2.07)
Unloading slope (% body weight/% stance phase duration)	−5.30 (1.66)	−5.89 (1.87)	−6.26 (2.10)	−6.91 (2.34)	−7.31 (2.71)	−7.41 (2.64)	−7.65 (2.79)	−7.75 (2.92)	−8.02 (3.02)

^a^Fmean_stance_: the mean force over the entire stance phase.

^b^Fmean_load_: the mean force between the start of the loading phase and Fz2.

^c^Fmean_mid_: the mean force between Fz2 and Fz4.

^d^Fmean_unload_: the mean force between Fz4 and the end of the unloading phase.

^e^Fz2: the first maximum.

^f^Fz3: the minimum.

^g^Fz4: the second maximum.

**Table 3:** Linear regression slopes and R2 values for each of the curves shown in Figure 3.

	Linear regression slope (%)	*R* ^2^
Fmean_stance_^a^ (% body weight)	−0.002	0.001
Fmean_load_^b^ (% body weight)	0.930	0.943
Fmean_mid_^c^ (% body weight)	0.116	0.527
Fmean_unload_^d^ (% body weight)	0.807	0.909
Fz2^e^ (% body weight)	−0.926	0.907
Fz3^f^ (% body weight)	0.367	0.483
Fz4^g^ (% body weight)	0.894	0.952
Loading slope (% body weight/% stance phase duration)	−2.109	0.910
Unloading slope (% body weight/% stance phase duration)	0.900	0.935

^a^Fmean_stance_: the mean force over the entire stance phase.

^b^Fmean_load_: the mean force between the start of the loading phase and Fz2.

^c^Fmean_mid_: the mean force between Fz2 and Fz4.

^d^Fmean_unload_: the mean force between Fz4 and the end of the unloading phase.

^e^Fz2: the first maximum.

^f^Fz3: the minimum.

^g^Fz4: the second maximum.

## Discussion

### Principal Results

This study identified characteristic changes when walking with an uphill or downhill slope in insole plantar pressure data of healthy participants. The most pronounced changes with treadmill slope were found in the loading slope of the curve. A typical combination of changes in several parameters was reported that defines uphill and downhill walking and may be used for annotation and correction when analyzing such data. These changes in the trajectory of the force curve with different surface slopes relative to the force vector of Earth’s gravity are related to changes in plantar load distribution. When walking downhill, Fz2 was found to be higher compared to when walking uphill, which is caused by the more pronounced force transfer through the heel of the foot, followed by a lower second maximum due to the even lower surface at push-off.

While patient-related factors, such as curve characteristics related to body size, muscle power, degenerative disease, etc, would remain constant throughout an insole measurement, fatigue-related changes [15] may increasingly appear and then stay toward the later stages of a recording of a walking bout. Additionally, age, body height, body weight, BMI, and handgrip strength were shown to cause characteristic changes in the plantar pressure force curve, that would usually only change on a long-term scale [16]. In contrast, as shown in the present data set, walking on slopes leads to temporary and characteristic changes in specific properties of the stance-phase curve. Changes over time in the identified parameters should thus be considered and correctly interpreted when studying long-term field gait data collected via insoles. To analyze the healing process, that is, after an injury, slow changes in parameters would be expected, and a trend toward what is considered normal over several weeks [17]. Short-term changes over minutes or hours would thus not be explainable by the healing progress and should have a different cause. In addition, the asymmetry between the legs should slowly decrease throughout healing [18]. When walking on a slope, asymmetry could also be affected, if the injury causes increasing problems such as pain when walking uphill or downhill. It is also recommendable to identify the characteristics of walking with walking aids, such as crutches, to be able to classify the nature of the observed changes and the treatment stage better.

### Limitations

Effects of walking speed were not analyzed in this study, even though it is known that lower extremity joint loading is affected by varying step length and cadence during graded uphill and downhill walking [19]. These parameters, however, do not seem to be necessary to successfully annotate gait data obtained by insoles. For participant or patient convenience, it would be desirable if insoles did not need to be combined with further devices or wearables. The present data suggest that at least the identification of walking on slopes does not require further sensors. It is also known that kinematic, kinetic, and electromyographic parameters differ between treadmill walking and overground gait, while spatiotemporal, kinematic, kinetic, electromyographic, and energy consumption outcome measures are largely comparable [20]. Another limitation of this study is that the parameters analyzed here can only be used when a regular gait curve is present. If this is not the case, other methods need to be applied, that is, machine learning for step detection and segmentation or the analysis of further parameters, possibly slopes and averages, or differences between individual sensors [21]. Differences between the 16 sensors embedded in each insole were not analyzed in this study and could be assessed in the future, for example, to distinguish between ground types (gravel, sand, etc). Another limitation is that the present characteristic changes that were assessed in healthy participants may differ for patients with gait disorders, depending on their disease or injury type. It will therefore be important to collect longitudinal data on different slopes from patients with defined diseases and injuries throughout the healing process or throughout different disease stages. These studies would serve to identify if the reported findings are valid also for patients, and for which patient groups this is true.

### Use of Wearables in Patients

The insole technology and present data may be valuable in real-world settings when investigating changes in mechanical properties during walking, that is, in occupational health research, sport and exercise science, for urban planning, and to plan inclusive architecture. For instance, the global average slope of urban areas is about 3.70° [22]. Wearables such as pressure insoles are increasingly used to study gait and movement, as well as for fall detection, fall classification, and fall risk assessment in the daily life of patients, and furthermore for lifestyle and health monitoring [1,3,23-27]. Long-term monitoring, especially if combined with additional sensors, may produce large amounts of data that require advanced strategies for analyses. Apart from regression statistics, among the options is the use of machine learning algorithms trained with annotated data for pattern recognition [24,26]. For longer-term monitoring of patients, it would be desirable if such algorithms were trained to identify various key activities of daily life that might indicate the level of healing progress. For example, when a patient with a tibial fracture is capable of cycling again, this is likely an indication for advances in the healing process. It would also be of interest to identify risky behavior, possibly leading to excessive forces, and to warn the patient by giving, for example, an audible or haptic warning signal. To guarantee meaningful data interpretation, machine learning may be combined with conventional regression-based analyses, such as the ones proposed in this paper to best tackle data complexity. Furthermore, prediction algorithms could be implemented for falls and diseases that enable more refined individual recommendations. Ideally, such interventions would be based on live data analyses. Limitations in the computing power of small wearable devices can increasingly be mitigated by both algorithmic optimization techniques in machine learning, such as dimensionality reduction, reservoir computing, and network pruning, as well as hardware innovations [27,28]. In the near future, such advances will likely allow real-time feedback based on data from various sources combined [29,30]. Alternatively, extracting decision-making systems (symbolic artificial intelligence), such as threshold-based methods, might offer an immediate route to real-time feedback.

### Sensors in Orthoses and Implants

Apart from insoles, very similar data might be collected from mechanical sensors embedded in orthoses [31] or implants [32]. Potentially, walking on a slope in these recordings changes the data in similar ways as described here. It would be highly desirable if patients did not need to use separate wearables such as insoles anymore, but if orthoses and implants had sensors embedded, not only to monitor healing progress but also to identify healing problems or complications and the need for surgical revision [33]. If similar load data could be collected by sensors in artificial hip or knee joints, or potentially even by plates or nails that stabilize bone fractures, recovery regimen could be monitored continuously and advice given on time [34]. Alarms could go off if forces exceeded certain thresholds or if live pattern analyses revealed unfavorable patterns known to be associated with exceeding forces or problems. As these developments seem to have a high potential with regard to rehabilitation and postoperative treatment, data analyses of insole data should be further studied and ideally, details such as algorithms and characteristics should be published to enable for the further development and widespread application of the named interventions.

### Conclusions

Characteristic changes in the plantar-pressure stance phase curve were identified, which reflect uphill and downhill walking. Automated annotation and continuous analyses of gait data via wearables could enable improved rehabilitation and feedback systems for prevention and treatment. A combination of traditional regression statistics embedded in heuristics combined with artificial intelligence methods may yield the best results.

## Appendix

Multimedia Appendix 1 Data file.

## Abbreviations

Fmean_load_: the mean force between the start of the loading phase and Fz2
Fmean_mid_: the mean force between Fz2 and Fz4
Fmean_stance_: the mean force over the entire stance phase
Fmean_unload_: the mean force between Fz4 and the end of the unloading phase
Fz2: the first maximum
Fz3: the minimum
Fz4: the second maximum
